# The Register

**Published:** 1881-10

**Authors:** Jno. Thad. Johnson, James B. Baird


### Editorial.

### THE REGISTER.

The undersigned, in assuming the editorial management of the ATLANTA MEDICAL REGISTER, deem it appropriate to announce that it shall be conducted as an absolutely independent journal.

It is not, and it shall never become, the organ or the instrument of any faction, firm, organization or instiution. It is designed to be a medium of communication and acquaintanceship among medical men ; and a means for conveying information, disseminating knowledge, promoting the truth, upholding the right and cementing the ties of professional brotherhood.

Toward the accomplishment of these aims we pledge our zealous care, and we earnestly invoke the aid and cooperation of our brethren everywhere.

JNO. THAD. JOHNSON,
JAMES B. BAIRD.

